# Protective effects of GuanXinNing tablet (GXNT) on diabetic encephalopathy in zucker diabetic obesity (ZDF) rats

**DOI:** 10.1186/s12906-023-04195-2

**Published:** 2023-10-27

**Authors:** Yajing Li, Jiaojiao Chen, Haiye Tu, Quanxin Ma, Mulan Wang, Jie Chen, Minli Chen

**Affiliations:** 1 The Department of Biopharmaceutical Technology, Zhejiang Institute of Economics and Trade, Hangzhou, 310018 China; 2 Institute of Comparative Medicine, Experimental Animal Research Center, Zhejiang Chinese Medical University, Hangzhou, 310053 China; 3 Hangzhou Institute for Advanced Study, UCAS, Hangzhou, 310012 China; 4 School of Pharmaceutical Sciences, Zhejiang Chinese Medical University, Hangzhou, 310053 China; 5 The Department of Medicine, Chiatai Qingchunbao Pharmaceutical Co., Ltd, Hangzhou, 310053 China; 6 Department of Vasculocardiology, The First Affiliated Hospital of Zhejiang Chinese Medicine University, Hangzhou, 310006 China

**Keywords:** GuanXinNing Tablet (GXNT), Diabetic Encephalopathy (DE), Zucker Diabetic fatty (ZDF) rats, Neuroprotection, Endothelial protection, Glycolipid Metabolism Regulation

## Abstract

**Background:**

Diabetic encephalopathy (DE) is a complication of diabetes that leads to cognitive and behavioral decline. Utilizing safe and effective complementary and alternative medications for its management is a wise choice. Previous studies have shown that GuanXinNing Tablet (GXNT), an oral preparation primarily derived from two Chinese herbs, *Salvia miltiorrhiza Bge*. and *Ligusticum chuanxiong Hort*., exerts a beneficial neuroprotective effect. In this study, we explored the protective effects of GXNT on DE in male Zucker diabetic fatty (ZDF) rats induced by a high-fat diet, aiming to ascertain its significance and potential mechanisms.

**Methods:**

ZDF rats were induced to develop type 2 diabetes (T2DM) with DE by a high-fat diet and treated with GXNT for 8 weeks until they were 20 weeks old. Throughout the experiment, the animals’ vital parameters, such as body weight, were continuously monitored. Cognitive function was evaluated using the Y maze test. Biochemical kits were employed to analyze blood glucose, lipids, and vascular endothelial-related factors. Cerebrovascular lesions were assessed using magnetic resonance angiography (MRA) imaging. Brain lesions were evaluated using hematoxylin and eosin (H&E) staining and ultrastructure observation. IgG and albumin (ALB) leakage were detected using immunofluorescence.

**Results:**

GXNT demonstrated an enhancement in the overall well-being of the animals. It notably improved cognitive and behavioral abilities, as demonstrated by extended retention time in the novel heterogeneous arm during the Y-maze test. GXNT effectively regulated glucose and lipid metabolism, reducing fasting and postprandial blood glucose, glycated hemoglobin (HbA1c), and total cholesterol (TC) levels. Additionally, it exhibited a protective effect on the vascular endothelium by reducing the serum TXB_2_/PGI_2_ ratio while elevating NO and PGI_2_ levels. Moreover, GXNT ameliorated stenosis and occlusion in cerebral vessel branches, increased the number of microvessels and neurons around the hippocampus, and improved microvascular occlusion in the cerebral cortex, along with addressing perivascular cell abnormalities. Immunofluorescence staining showed a decrease in the fluorescence intensity of IgG and ALB in the cerebral cortex.

**Conclusions:**

GXNT demonstrated a highly satisfactory protective effect on DE in ZDF rats. Its mechanism of action could be based on the regulation of glucolipid metabolism and its protective effect on the vascular endothelium.

**Supplementary Information:**

The online version contains supplementary material available at 10.1186/s12906-023-04195-2.

## Introduction

As a serious disease affecting human health, the incidence and prevalence of Diabetes mellitus are rapidly increasing. The International Diabetes Federation estimated that 536.6 million people lived with diabetes (diagnosed or undiagnosed) in 2021, and this number was projected to increase by 46%, reaching 783.2 million by 2045 [1, 2]. DE is one of the most serious and common complications of diabetes, resulting in a high rate of disability and posing a severe threat to human health [3]. DE encompasses intracranial macrovascular and microvascular diseases, where atherosclerosis is a primary pathological change in intracranial great vessel lesions [4]. Intracranial microvascular lesions are typically characterized by thickening of the microvascular basement membrane, microvascular thrombosis, and microcirculation disorders [5].

According to epidemiological investigations, diabetic patients face a 4 to 10 times higher risk of cerebrovascular disease compared to non-diabetic patients, making it a significant contributor to diabetic patient mortality [6]. Due to the absence of a gold standard for DE diagnosis, its onset remains unclear. Factors such as blood glucose and lipid disorders, brain microcirculation disruptions, thickening of the microvascular basement membrane, increased capillary permeability, elevated blood viscosity, oxidative stress, vascular and blood-brain barrier damage, Aβ amyloid deposition, and neuronal tangles are all considered etiological factors, pathogenic mechanisms, and risk factors for DE progression [7–9]. Therefore, understanding the risk factors of DE and early detection and control of its occurrence and progression are crucial.

Currently, controlling blood glucose levels is the fundamental approach in drug treatment for DE [10, 11]. For instance, metformin (Met), commonly used in the management of diabetes and its complications, can lower blood glucose levels, promote weight loss, and control hyperinsulinemia [12, 13]. Additionally, the assistance of G protein-coupled receptors and anti-inflammatory and antioxidant drugs is needed [14–17]. However, these drugs often have a singular mode of action and are accompanied by adverse reactions in the hepatobiliary and gastrointestinal systems. Traditional Chinese medicine (TCM) exhibits multi-target, multi-direction, and multifaceted pharmacological characteristics [18–21]. Hence, researching TCM preparations with clear efficacy, defined components of action, and explicit mechanisms for preventing and treating DE is important.

GXNT primarily consist of extracts from *Salvia miltiorrhiza* and *Ligusticum chuanxiong*, is used to treat coronary heart disease and angina pectoris (chest pain and heartache) [22–25]. It is a new formulation derived from the same components as GuanXinNing injection but with different dosage forms and administration routes [26–28]. The pharmacological activity of GXNT is the result of the synergistic action of all components. Previous studies have confirmed that GXNT possesses neuroprotective, blood circulation-promoting, lipid peroxidation-inhibiting, free radical scavenging-enhancing, and vascular endothelial cell membrane-stabilizing effects [29–32]. It can inhibit drug-induced thrombosis in zebrafish, with its effect being related to reducing free radical formation, inhibiting peroxidation, and inhibiting platelet aggregation [33].

ZDF rats with spontaneous Type 2 diabetes, which exhibit metabolic syndromes such as obesity, insulin resistance, lipid disorders, and abnormal glucose tolerance, closely mimic the pathogenesis of human T2DM. They are frequently used to study T2DM and its complications, such as diabetic nephropathy and diabetic retinopathy [34, 35]. The occurrence of DE symptoms is associated with aging and a sustained high-fat diet in animals. In this study, 8-week-old ZDF rats were fed a high-fat diet to induce typical T2DM symptoms. Based on this, the occurrence of DE was assessed in various aspects, including general physiological signs, blood biochemistry, cognitive and behavioral abilities, and histological examination.

Previous studies have lacked experimental research on the effects and mechanisms of GXNT or its components on DE. Therefore, this study aimed to address an intriguing question: whether GXNT has a protective effect against DE in ZDF rats induced by a high-fat diet. Furthermore, if GXNT does provide protection, does its regulation of glucose and lipid metabolism, as well as its vascular endothelial protective mechanisms, play a role in this protective effect?

## Materials and methods

### Drug preparation

GXNT (GuanXinNing tablet, abbreviated as GXNT, with a raw drug dosage of 12.8 g·g^− 1^) was supplied by Chiatai Qingchunbao Pharmaceutical Co., Ltd. (Hangzhou, China). The primary components of GXNT are derived from *Salvia miltiorrhiza Bge*. and *Ligusticum chuanxiong Hort*. The main active ingredients identified in GXNT include salvianolic acid B, ferulic acid, rosmarinic acid, tanshinol, chlorogenic acid, caffeic acid, and salvianolic acid A. The quality of GXNT is maintained based on the content of these specific components. Met (Metformin hydrochloride tablets, abbreviated as Met) with specifications: 0.5 g/tablet * 20 tablets, from Sino-American Shanghai Shi Guibao Pharmaceutical Co., Ltd. In terms of administration, both GXNT and Met were dissolved with 0.9% normal saline to reach the desired concentration.

### Establishment and intervention of ZDF rat model for T2DM with DE

ZDF rats, characterized by a missense mutation in the leptin receptor gene [36], and control animals were studied at the Experimental Animal Research Center, Zhejiang Chinese Medical University (Zhejiang, China), possessing the certificate number SYXK (ZHE) 2013 − 184. Thirty-two male ZDF rats, aged 8 to 9 weeks, were sourced from Beijing Vital River Laboratory Animal Technology Co., Ltd. with the certificate number SCXK (JING) 2016-006. These rats were individually housed under a regular light cycle in the animal facility and were provided with a control diet 5008 (Lab Diets, Purina # 5008), containing nutrient components such as 23.0% protein, 6.5% total fat and 4.0% crude fiber, throughout the study. The rats had unrestricted access to both food and water. Our study was submitted to and approved by the Institutional Animal Care and Use Committee (IACUC) of Zhejiang Chinese Medical University (Approval No. IACUC-20180502-01). The experiment was carried out in strict accordance with the international rules and regulations of laboratory animal ethics. The study was reported in accordance with ARRIVE guidelines.

## Grouping and treatment

**Fig. 1:**
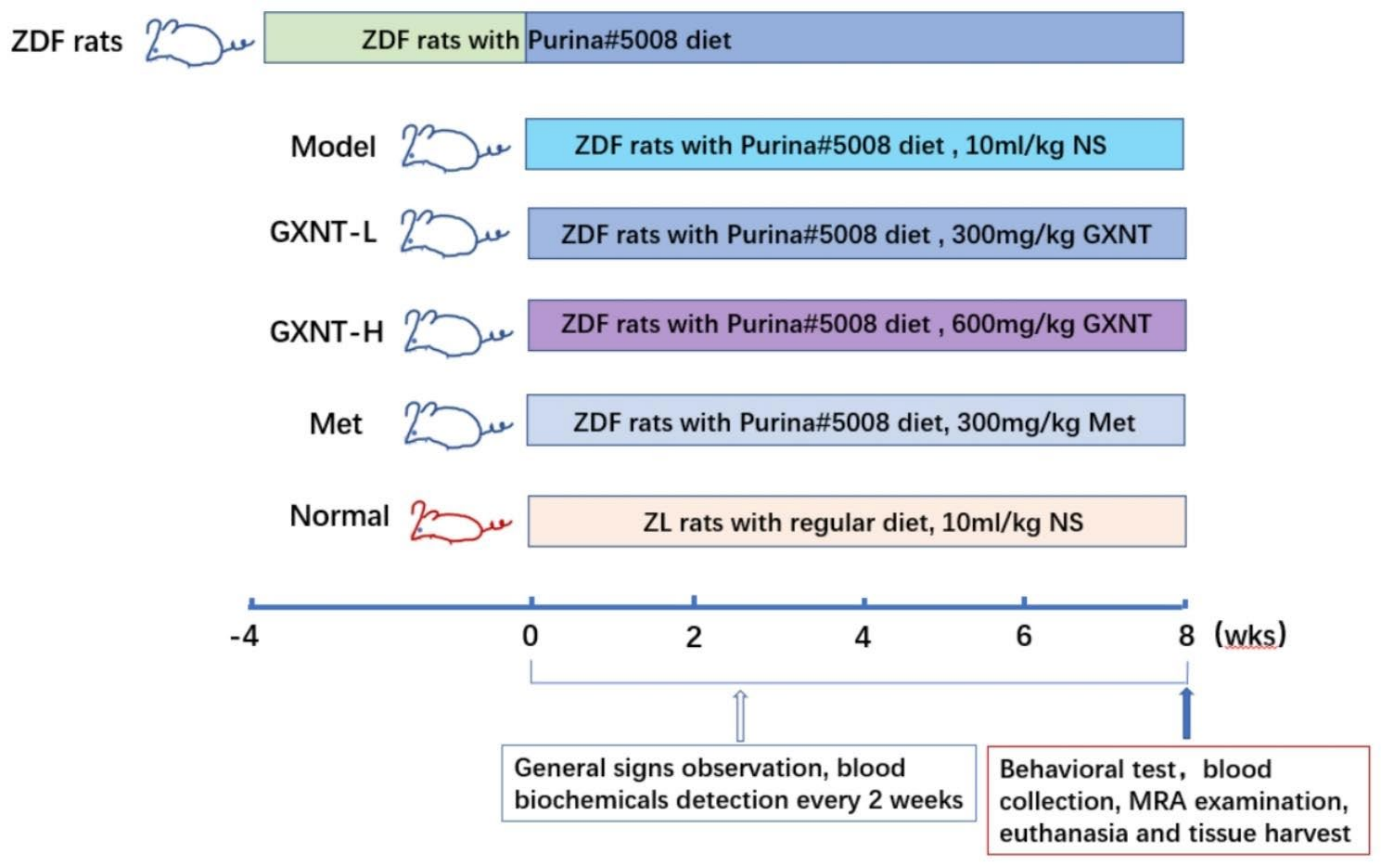
Experimental procedure overview. This schematic diagram outlines the experimental methodology. After 4 weeks of being on a high-fat diet (Purina # 5008), a ZDF rat model for T2DM with DE was established and categorized for treatment, using ZL rats as the normal control. The ZDF rats were continuously fed a high-fat diet, while the ZL rats were on a normal diet. The drug administration was a daily routine. Throughout the experiment, various parameters were measured. Body weight, food and water intakes, and blood biochemicals such as fasting and postprandial glucose (n = 8) were assessed every 2 weeks. Additionally, HbA1c,TC, and TG (n = 8) were measured every 4 weeks. At the 8th week (animal 20 weeks of age), the Y-maze test (n = 6) was conducted to evaluate cognitive behavior, and MRA (n = 2) was utilized for cerebrovascular imaging observations. Subsequently, blood samples were taken to detect glucose, TC, TG, HbA1c, NO, PGI_2_, and TXA_2_ (n = 8) before sacrifice. Brain tissue was then collected for histopathological observation (n = 8) after euthanasia

Thirty-two male ZDF rats, aged 8 to 9 weeks, were initially fed the Purina #5008 diet. After 4 weeks of this diet, the rats underwent a 10- to 12-hour period of water deprivation. Blood samples were then collected from beneath the jaw to determine serum glucose, TC, and TG levels. Based on body weight and blood glucose levels, the ZDF rats were categorized into four groups: Model, GXNT low-dose, GXNT high-dose, and Met groups, each consisting of eight rats. Additionally, eight Zucker lean (ZL) rats from the same age were fed an regular diet, constituting the normal group. During the experiments, there were two ZDF rats per cage and four ZL rats per cage. In the GXNT low-dose and high-dose groups, ZDF rats were orally administered GXNT at doses of 300 mg/kg and 600 mg/kg, respectively. The Met group was orally administered Met at 300 mg/kg (150 mg/kg twice a day). The model group and normal group were orally administered 10 mL/kg of normal saline daily. The experiment lasted for 8 consecutive weeks until the animals reached 20 weeks of age. Throughout the experiment, various parameters related to the rats’ well-being and health were observed, including feeding, drinking, urination, defecation, behaviors, and mental states. And the body weight, feed intake, and water intake were recorded. The doses of GXNT and Met used in this study were determined based on the standard body surface area normalization method, using a conversion factor of 7 to translate the human dosage of GXNT (4560 mg/60 kg/d) and Met (2550 mg/60 kg/d) to the equivalent dose for rats [37]. Data on animal body weight, food intake, and water intake were collected every 2 weeks (n = 8). Fasting blood glucose and postprandial blood glucose were measured every 2 weeks (n = 8). HbA1c, TC, and TG were measured every 4 weeks (n = 8). At the 8th week of the experiment, when the animals were 20 weeks old, Y-maze testing was conducted (n = 6). Brain vascular MRA imaging was performed (n = 2). At the end of the experiment, animals were euthanized, and blood samples were collected to analyze endothelial factors NO, PGI_2_, and TXB_2_ (n = 8). Brain tissue sections were prepared for H&E staining, ultrastructural observation, and IgG and ALB fluorescence immunostaining (n = 8). The experimental design process is illustrated in Fig. 1.

### Y maze test

The Y maze test was divided into two phases: a training period and a testing period. The rats were initially placed in arm A (the starting arm). During the training phase, arm B (the new and distinct arm) was blocked by a baffle, and the rats were allowed to freely explore A and C (other arms) for 10 min before being returned to the IVC cage. The testing phase took place an hour later. The baffle blocking arm B was removed, enabling the rats to move freely across all three arms for 5 min. The duration the rats spent in the B arm within the 5-minute period was recorded using a camera. Their cognitive abilities were assessed by observing and calculating the duration of their stay in arm B.

### Measurement of blood biochemical parameters

Blood biochemical parameters were measured during the 4th and 8th weeks of administration using the corresponding kits (Ningbo Medical System Biotechnology Co. Ltd., China). Following a 10- to 12- hour period of water deprivation, 0.5 mL of blood was collected from beneath the jaw. Serum was separated, and serum glucose, TC, and TG levels were measured by an automatic biochemical analyzer (7020, ITACHI, Japan). Simultaneously, 200 µL of whole blood was collected, and the level of HbA1c was measured using an HbA1C analyzer (Trinity Biotech, Ireland). Additionally, serum levels of markers associated with vascular endothelial function (NO, PGI_2_, and TXB_2_) were measured following the respective instructions of kits from Jiancheng Bioengineering Institute, Nanjing, China.

### MRA analysis

Following 8 weeks of treatment, 2 rats from each group were randomly chosen for MRA scans. Signals were acquired using a superconducting magnetic resonance scanner (GE Discovery MR 750, USA) equipped with a specialized coil for rats (Shanghai Chenguang Medical Technology Co., Ltd., China). MRA scans were conducted using 3D time-of-flight (3D TOF) sequences with the following parameters: repetition time (TR) = 31 ms, echo time (TE) = 4.9 ms, flip angle = 30°, slice thickness = 0.6 mm, field of view (FOV) = 80 mm×100 mm, matrix = 384 × 256 (frequency × phase) [38].

### Histopathological observation and immunofluorescence staining

After 8 weeks of treatment, animals were deeply anesthetized with an intraperitoneal injection of 3% pentobarbital sodium solution (0.15 mL/100 g, Merck &Co., USA) before euthanasia, and the brain tissues were carefully dissected for histopathological examination. The collected tissue samples were fixed in 4% paraformaldehyde, embedded, and sectioned to a diameter of 4 μm. Subsequently, sections were stained with hematoxylin and eosin (H&E) to observe the morphological changes in the microvessels and neurons around the hippocampus. The sections were scanned using the NanoZoomer digital pathological section scanner (NanoZoomer 2.0-RS, Hamamatsu, Japan). Six sections per rat were chosen for analysis. The number of intact neurons (cells with round and palely stained nuclei) and microvessels (blood vessels with one to two flattened endothelial cell nuclei) in the hippocampal dentate gyrus (DG) area was blindly counted by a second investigator. Furthermore, the ultrastructure of cortical microvessels and synapses in rats was observed by transmission electron microscopy. Briefly, the cortex of the brain tissue was swiftly cut into small pieces of about 1mm^3^ and fixed in 2.5% glutaraldehyde. After proper processing involving dehydration, immersion, embedding, and sectioning, staining with lead citrate was performed. The sections were then examined using a transmission electron microscope.

For immunofluorescence staining, after dewaxing, the sections were incubated with ALB antibody (1:200; Santa Cruz Corporation, USA; 100 µ L/piece, Lot number sc71605) and Biotin Goat anti-mouse IgG antibody (1:200; Immunoway, USA; 100 µ L/piece, Lot number RS0001) overnight at 4 °C. Following a wash, the sections were labeled with Alexa Fluor 488-conjugated goat anti-mouse IgG and Alexa Fluor 647-conjugated goat anti-mouse IgG (Abcam, UK) in the dark at 37 °C for 45 min. Subsequently, the sections were washed with PBS twice, fixed with DAPI fluorescent lamps, and stored in the dark at 4 °C. The NanoZoomer digital pathological section scanner (NanoZoomer 2.0-RS, Hamamatsu, Japan) was utilized to digitally scan the brain tissue sections, and image-pro Plus 6.0 software was employed to analyze the percentage of the stained area.

### Statistical analysis

Data were presented as means ± SEM. Variable analysis and visualization were carried out using GraphPad Prism 8.0 software (GraphPad Software Inc., USA). Prior to analysis, Bartlett’s test and the Kolmogorov-Smirnov test were used to test for homogeneity of the variances and normality of the response variables, respectively. All response variables passed tests of normality (Kolmogorov-Smirnov (distance) test: P > 0.10). For data analysis, repeated measures (RM) two-way analysis of variance (ANOVA), and parametric one-way ANOVA were performed. As not all variables met the assumption of homogeneity of variances (Bartlett’s test: P < 0.01), we used an ordinary one-way ANOVA test followed by Tukey’s multiple comparisons test when the data conformed to homoscedasticity and employed Welch’s ANOVA test followed by Dunnett’s T3 multiple comparisons test when heteroscedasticity was observed. In two-way ANOVA, the Geisser-Greenhouse correction was used to address heterogeneity of variances and ensure homogeneity of variances. Following the ANOVA, Tukey’s multiple comparisons test was conducted to assess group differences. Statistical significance was defined as p < 0.05. Sample size calculation and power analysis were performed using G* Power software (version 3.1.9.7; Heinrich-Heine University). For a one-way ANOVA model, assuming an effect size of 0.69 for behavioral assessment (n = 6), an effect size of 0.58 for biochemical assessment (n = 8), and an effect size of 0.58 for histological assessment (n = 8), with a desired power of 0.8 and a significance level of 0.05.

## Results

### Effects of GXNT on body weight, food intake, and water intake in T2DM ZDF rats with DE

**Fig. 2:**
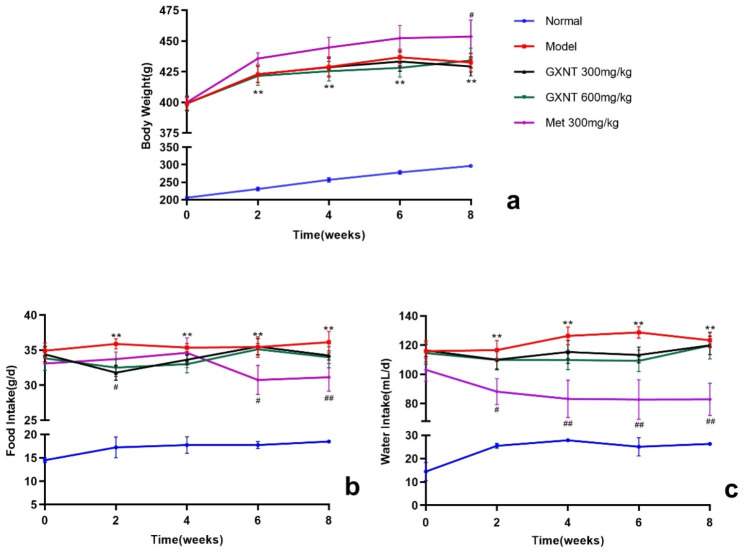
Effect of GXNT on body weight, food, and drink intakes in T2DM ZDF rats with DE. (**a**) depicts the impact of GXNT on body weight changes; (**b**) illustrates the effects of GXNT on average daily food intake; (**c**) presents the effects of GXNT on average daily water intake. GXNT, Guanxinning tablet; Met, metformin. Data are expressed as mean ± SEM, n = 8. * p < 0.05, **P < 0.01 vs. normal group (Zucker lean control rats); #P < 0.05, ##P < 0.01 vs. model group

Body weight serves as an indicator of the animals’ basic status. As shown in Fig. 2a, the body weight of ZDF rats in the model group significantly surpassed that in the normal group from the 2nd to the 8th week of administration (F _2.868, 20.08_ = 213.3; P < 0.01). However, there was no notable difference in body weight between the GXNT treatment groups and the model group (P > 0.05). Moreover, the rats in the Met group exhibited a higher weight compared to those in the model group after 2 weeks of Met treatment, and this difference became significant by the 8th week (P < 0.05).

Since food and water intakes are closely related to blood glucose and blood lipid levels, we closely monitored food and water intakes throughout the experiment. The findings revealed that, in comparison to the normal group, both food and water intakes of rats in the model group significantly increased (P < 0.01). Conversely, GXNT and Met treatments resulted in reductions in the food and water intakes of ZDF rats. Specifically, the food intake of the 300 mg/kg GXNT group was notably reduced compared to that of the model group at 2 weeks after administration (P < 0.05); in the Met group, a significant reduction was observed at 6 weeks and 8 weeks after administration (P < 0.05, P < 0.01). Furthermore, the water intake of the Met group was significantly reduced compared to that of the model group at 2–8 weeks after administration (P < 0.05, P < 0.01) (Fig. 2b, c).

### Effect of GXNT on the Y maze test in T2DM ZDF rats with DE

**Fig. 3:**
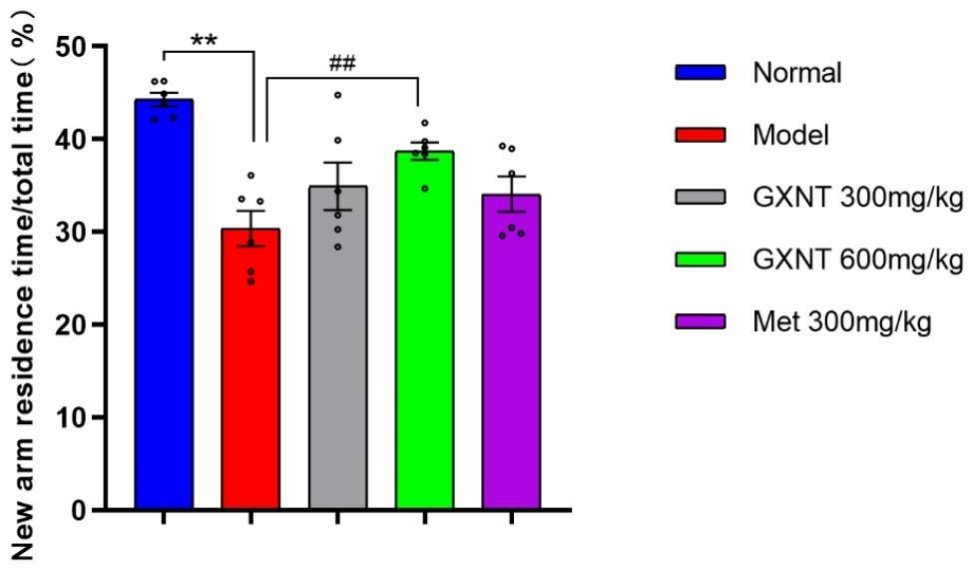
Effect of GXNT on the Y maze test in T2DM ZDF rats with DE. The rats’ cognitive ability was assessed by observing and calculating the duration of their stay in the B-arm, and the data is presented as a ratio of the time spent in the new arm to the total time spent. GXNT, Guanxinning tablet; Met, metformin. Data are expressed as mean ± SEM, n = 8. * p < 0.05, **P < 0.01 vs. normal group (Zucker lean control rats); #P < 0.05, ##P < 0.01 vs. model group

To evaluate the cognitive and behavioral abilities of the experimental rats, the Y maze experiment was conducted. As shown in Fig. 3, the duration of stay in the new arm was significantly reduced in the model group compared to the normal group (F _4, 25_ = 9.162; P < 0.01). These results suggest that DE negatively impacts the cognitive abilities of the animals, diminishing their interest and capacity to explore new environments. In contrast, rats treated with the drug exhibited varying degrees of increased time spent in the new arm, with the 600 mg/kg GXNT group displaying a significant extension (P < 0.01). These findings indicate that GXNT treatment notably enhanced the cognitive and behavioral abilities of the animals.

### Effects of GXNT on blood glucose and HbA1c levels in T2DM ZDF rats with DE

**Fig. 4:**
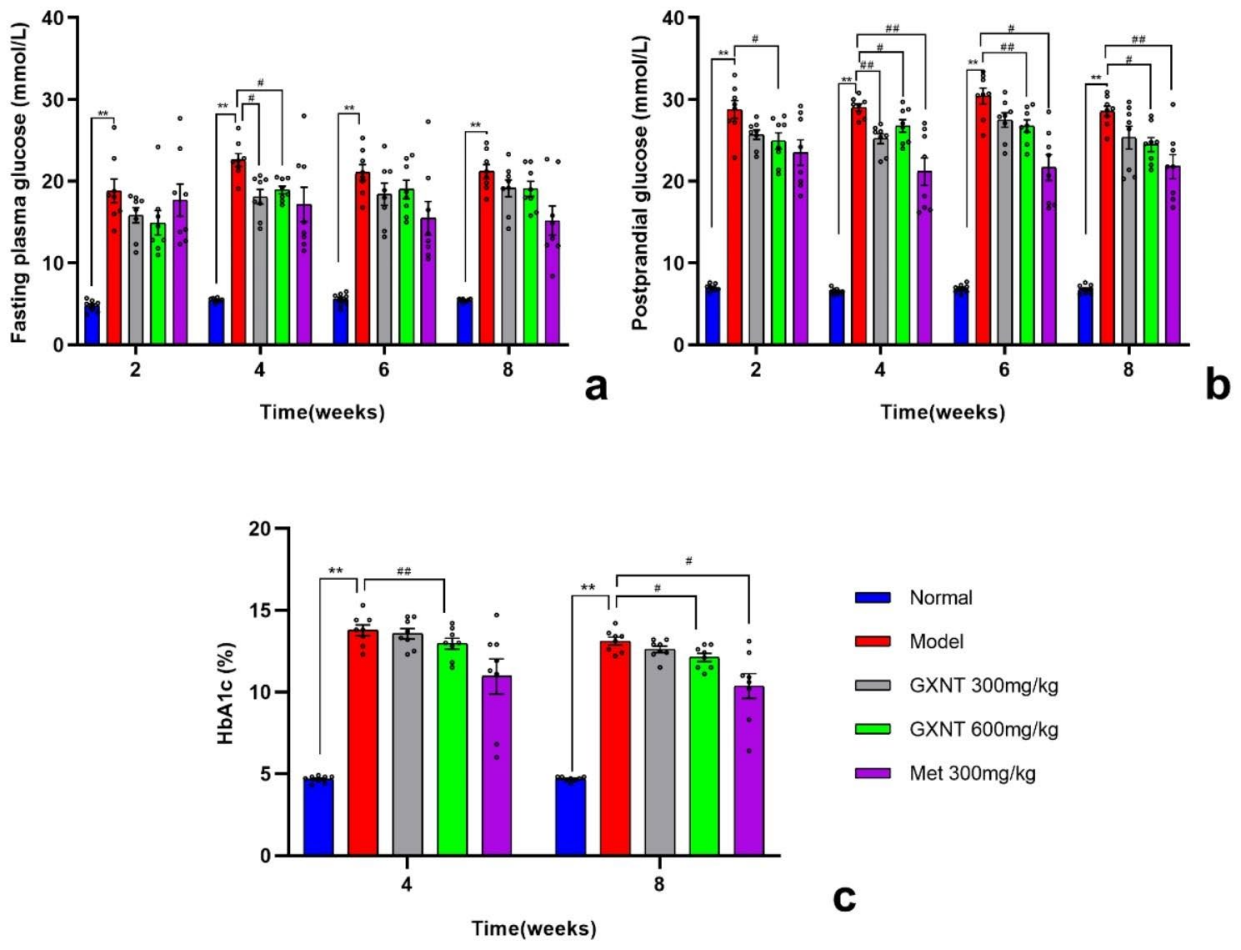
Effects of GXNT on fasting blood glucose, postprandial glucose, and HbA1c in T2DM ZDF rats with DE. (**a**) shows the effects of GXNT on the level of fasting blood glucose levels; (**b**) depicts the effects of GXNT on the level of postprandial glucose levels; (**c**) demonstrates the effects of GXNT on HbA1c levels. GXNT, Guanxinning tablet; Met, metformin. Data are expressed as mean ± SEM, n = 8. * p < 0.05, **P < 0.01 vs. normal group (Zucker lean control rats); #P < 0.05, ##P < 0.01 vs. model group

A repeated measures (RM) two-way ANOVA was conducted to assess the influence of the drug, time, and drug × time interaction on the levels of fasting blood glucose, postprandial blood glucose, and HbA1c in rats. The fasting blood glucose levels exhibited significant effects of drug (F _1.753, 12.27_ = 39.87; p < 0.001) and time (F _1.759, 12.31_ = 6.912; p < 0.05), with no significant effect of drug × time interaction (F _3.796, 26.57_ = 2.403; p > 0.05) (Fig. 4a). Postprandial blood glucose levels showed significant effects of drug (F _2.105, 14.73_ = 131.0; p < 0.001) and time (F _2.136, 14.95_ = 9.772; p < 0.01), but no significant effect of drug × time interaction (F _3.502, 24.52_ = 2.622; p > 0.05) (Fig. 4b). HbA1c levels also demonstrated significant effects of drug (F _1.339, 9.372_ = 78.26; p < 0.001) and time (F _1, 7_ = 21.83; p < 0.01), without a significant effect of drug × time interaction (F _2.065, 14.45_ = 1.929; p > 0.05) (Fig. 4c).

In comparison to the normal group, the model group displayed a significant increase in fasting and postprandial blood glucose levels (P < 0.01), as well as a significant increase in HbA1c levels (P < 0.01). As anticipated, GXNT, similar to the antidiabetic drug Met, influenced blood glucose levels in diabetic rats, resulting in a reduction in fasting blood glucose, postprandial blood glucose, and glycated hemoglobin levels, as depicted in Fig. 4a-c. Tukey’s multiple comparisons test demonstrated that both the 300 mg/kg and 600 mg/kg GXNT groups exhibited a significant decrease in fasting blood glucose levels compared to the model control rats at the 4th week of administration (p < 0.05, p < 0.01), as shown in Fig. 4a. Relative to the model group, both the GXNT and Met groups of rats showed a decreasing trend in postprandial blood glucose and HbA1c levels. Specifically, the 300 mg/kg GXNT group demonstrated a significant reduction in postprandial blood glucose levels at the 4th week after administration (p < 0.01). The 600 mg/kg GXNT group exhibited a significant decrease in postprandial blood glucose levels from the 2nd to 8th week after administration (P < 0.01, P < 0.05), and the Met group demonstrated a significant decrease in postprandial blood glucose levels from the 4th to 8th week after Met treatment (P < 0.01, P < 0.05), as shown in Fig. 4b. Regarding HbA1c levels, the 600 mg/kg GXNT group demonstrated a significant decrease compared to the model group at both the 4th and 8th weeks after administration (P < 0.01, P < 0.05). The Met group also exhibited a significant decrease compared to the model group at the 8th week after treatment (P < 0.05), as shown in Fig. 4c. Interestingly, based on the effects on postprandial blood glucose and HbA1c levels, it can be observed that the 600 mg/kg GXNT has a better long-term control effect on blood glucose, which is highly beneficial for controlling the development of DE.

### Effects of GXNT on blood lipids in T2DM ZDF rats with DE

**Fig. 5:**
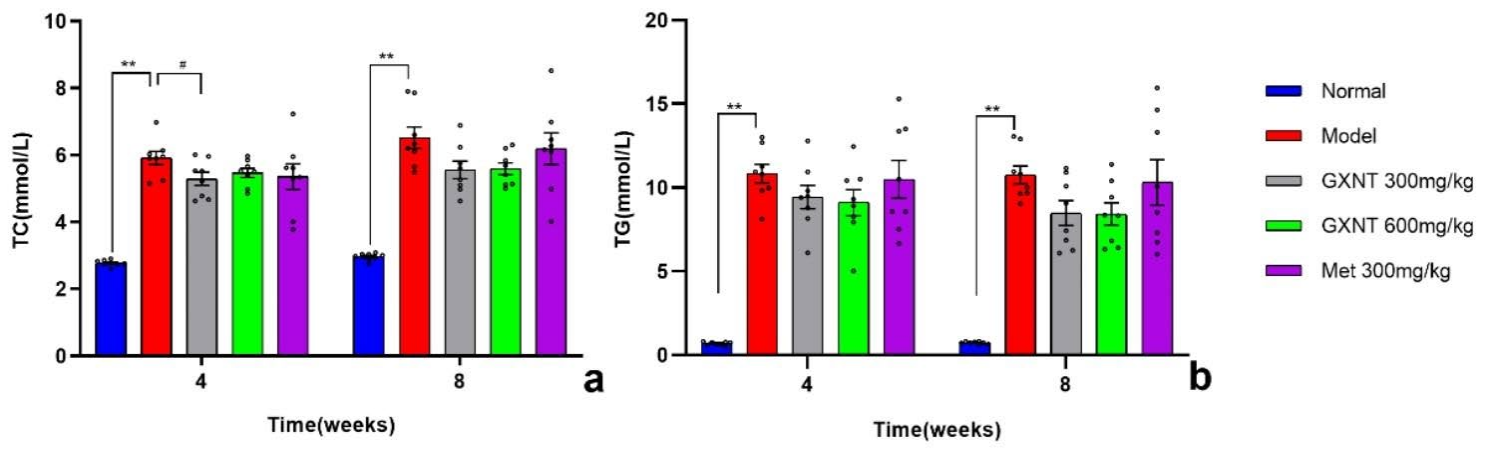
Effects of GXNT on blood lipid levels in T2DM ZDF rats with DE. (**a**) illustrates the effects of GXNT on TC levels; (**b**) displays the effects of GXNT on TG levels. GXNT, Guanxinning tablet; Met, metformin. Data are expressed as mean ± SEM, n = 8. * p < 0.05, **P < 0.01 vs. normal group (Zucker lean control rats); #P < 0.05, ##P < 0.01 vs. model group

Drug, time, and drug × time interaction effects on TC and TG levels in DE rats were evaluated by a two-way ANOVA. The levels of TC in the rats showed a significant effect of drug (F _1.856, 12.99_ = 29.40; p < 0.001), time (F _1, 7_ = 16.33; p < 0.01), and drug × time interaction (F _2.521, 17.65_ = 4.867; p < 0.05) (Fig. 5a). The levels of TG showed a significant effect of drug (F _4, 28_ = 36.72; p < 0.001), while there was no significant effect on time (F _1, 7_ = 1.626; p > 0.05) or drug × time interaction (F _4, 28_ = 0.2753; p > 0.05) (Fig. 5b). Tukey’s multiple comparison analysis revealed that, compared to the normal group, the levels of TC and TG in the model group rats were significantly increased (P < 0.01), indicating a lipid metabolism disorder in the DE model rats. Compared to the model group, the TC and TG levels in the GXNT and Met intervention groups showed varying degrees of reduction, with no significant differences observed at the 4th and 8th weeks (p > 0.05) for both 600 mg/kg GXNT and Met (Fig. 5a and b). However, the TC level in the 300 mg/kg GXNT group was significantly reduced at the 4th week (P < 0.05) (Fig. 5a). These findings suggest that GXNT has a certain regulatory effect on blood lipid abnormalities in T2DM rats, thereby reducing the occurrence of diabetes-related complications such as DE.

### Effects of GXNT on serum vascular endothelial factors in T2DM ZDF rats with DE

**Fig. 6:**
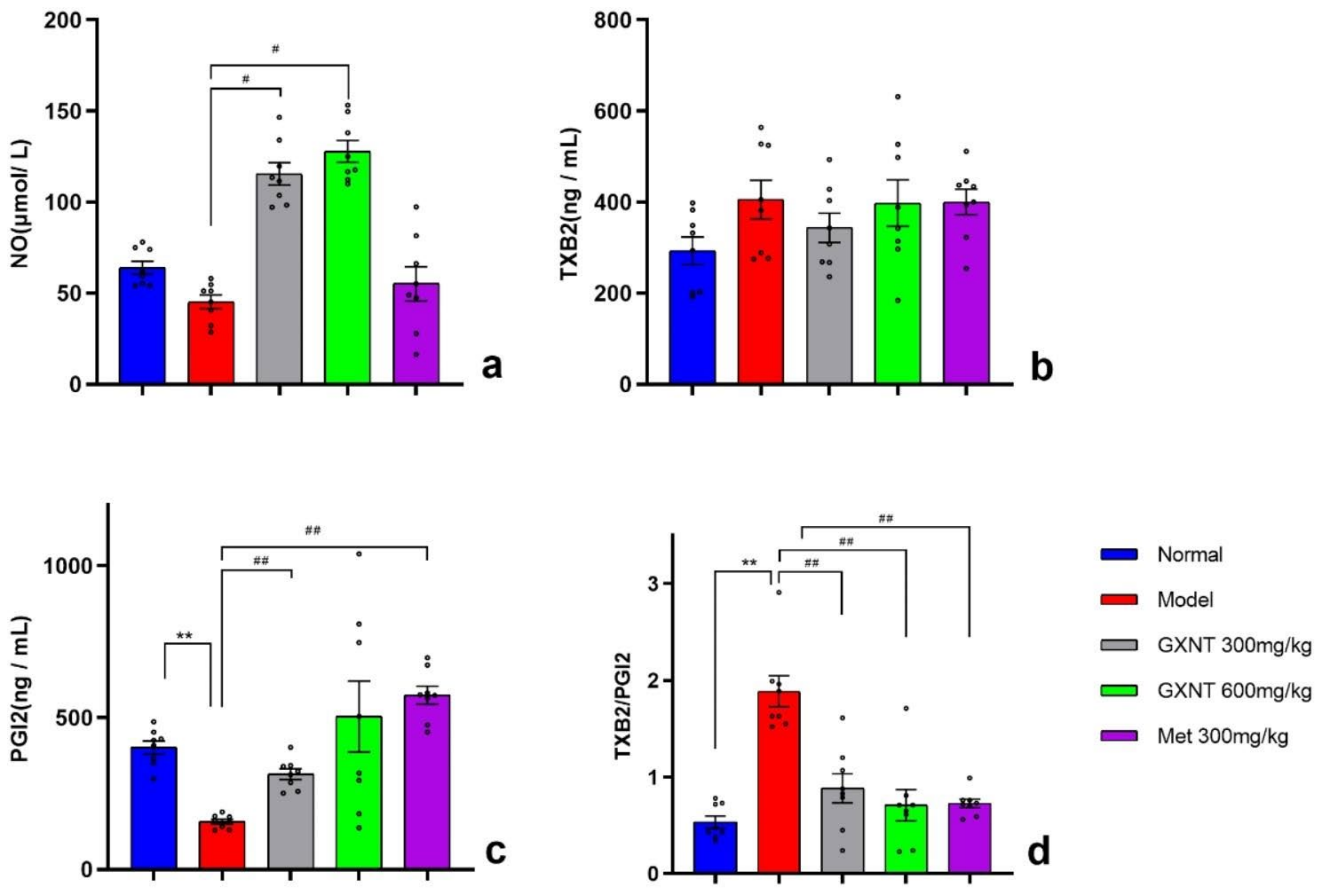
Effects of GXNT on serum vascular endothelial factors in T2DM ZDF rats with DE. (**a**) illustrates the effects of GXNT on NO levels; (**b**) demonstrates the effects of GXNT on TXB_2_ levels. (**c**) shows the effects of GXNT on PGI_2_ levels; (**d**) displays the effects of GXNT on the TXB_2_ /PGI_2_ ratio. GXNT, Guanxinning tablet; Met, metformin. Data are expressed as mean ± SEM, n = 8. * p < 0.05, **P < 0.01 vs. normal group (Zucker lean control rats); #P < 0.05, ##P < 0.01 vs. model group

GXNT significantly affected the levels of serum vascular endothelial factors in T2DM rats. One-way ANOVA showed that drugs significantly influenced serum NO levels (F _4, 35_ = 37.26; p < 0.001) (Fig. 6a), PGI_2_ content (W _4.000, 15.83_=69.29; p < 0.001) (Fig. 6c), and TXB_2_/PGI_2_ ratio (F _4, 35_ = 17.90; p < 0.001) (Fig. 6d), but had no significant effect on TXB_2_ levels (F _4, 35_ = 1.644; p > 0.05) (Fig. 6b). Tukey’s multiple comparisons test was used to compare the NO levels, TXB_2_ levels, and TXB_2_/PGI_2_ ratio among different groups. On the other hand, Dunnett’s T3 multiple comparisons test was employed to compare the PGI_2_ content between groups. The results revealed that, compared to the normal group, the serum PGI_2_ content was significantly decreased in the model control rats (P < 0.05) (Fig. 6c), and the ratio of TXB_2_/PGI_2_ was significantly increased (P < 0.01) (Fig. 6d). In comparison with the model group, rats treated with 300 mg/kg and 600 mg/kg GXNT showed a significant increase in serum NO level (P < 0.05) (Fig. 6a) and a significant decrease in the TXB_2_/PGI_2_ ratio (P < 0.01) (Fig. 6d). Rats treated with 300 mg/kg GXNT also exhibited a significant increase in PGI_2_ content (P < 0.01) (Fig. 6c). In the Met group, PGI_2_ content significantly increased (P < 0.01) (Fig. 6c), while the TXB_2_/PGI_2_ ratio significantly decreased (P < 0.01) (Fig. 6d). When serum NO and TXB_2_ levels showed no significant difference but PGI_2_ levels significantly decreased, resulting in an increased TXB_2_/PGI_2_ ratio, it may indicate higher platelet activity and weakened vasodilation, leading to an increased risk of thrombosis in the DE model rats. The effects of GXNT on reducing the TXB_2_/PGI_2_ ratio could be beneficial in maintaining normal blood circulation and preventing further development of DE.

### GXNT ameliorated cerebral vascular status in T2DM ZDF rats with DE

**Fig. 7:**
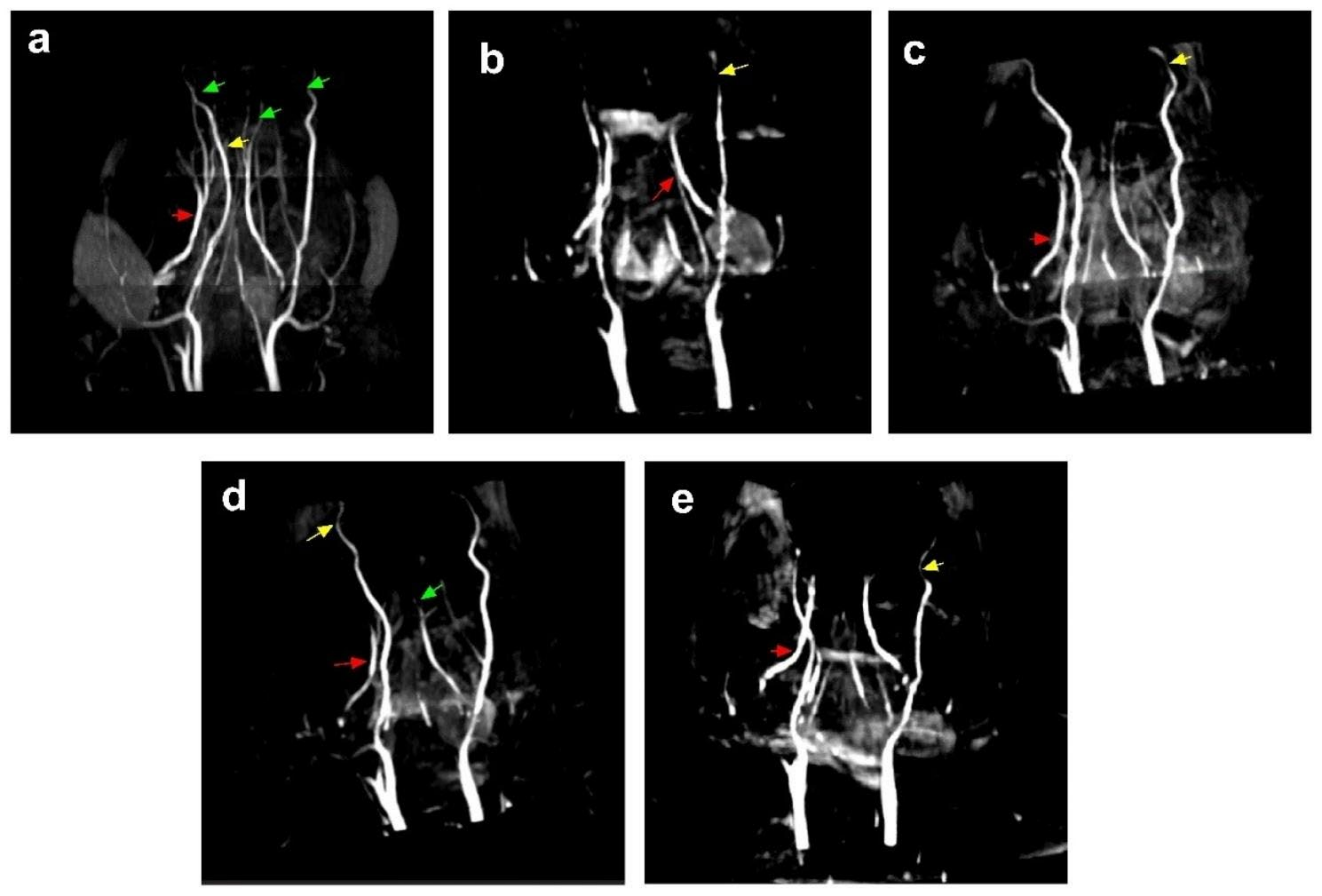
GXNT ameliorated cerebral vascular imaging in T2DM ZDF rats with DE. Cerebral vascular status in experimental rats was observed and assessed by MRA. Two rats from each group were randomly selected for MRA scans. (**a**) illustrates the intracerebral vascular status in normal rats: the vertebral artery was strong, and the intracerebral artery was clearly visible, without stenosis or occlusion. (**b**) illustrates the intracerebral vascular status in model rats: the vertebral artery was strong, but the intracerebral artery showed segmental stenosis, fewer distal branches and poor development, severe occlusion such as interrupted blood flow, and no distal development. (**c**) illustrates the intracerebral vascular status in rats treated with GXNT 300 mg/kg: the vertebral artery was strong, with partial cerebral artery occlusion and branch artery stenosis. (**d**) illustrates the intracerebral vascular status in rats treated with GXNT 600 mg/kg: the vertebral artery was strong; no occlusion of the internal cerebral artery; mild stenosis of the branch artery. (**e**) illustrates the intracerebral vascular status in rats treated with Met 300 mg/kg: the vertebral artery was strong, but the intracerebral artery showed segmental stenosis, fewer distal branches, and poor development. The vertebral artery is marked by red arrows; the intracerebral artery is marked by yellow arrows; and the branch artery is marked by green arrows

GXNT demonstrated the potential to ameliorate cerebral vascular status in T2DM ZDF rats with DE. MRA was employed to visualize and assess the cerebral vascular status. In the normal group, the vertebral artery exhibited strength and clarity, and the intracerebral artery was clearly visible without stenosis or occlusion (Fig. 7a). Conversely, the model control rats showed segmental stenosis in the intracerebral artery, fewer distal branches, poor development, and severe occlusions like interrupted blood flow and limited distal development, while the vertebral artery visualization was similar to the normal control group (Fig. 7b). Following GXNT administration, notably at 600 mg/kg dosage, the intracerebral artery occlusion was greatly improved, and while some branch artery stenosis persisted, it was notably less severe than in the model group (Fig. 7d). On the other hand, the Met group, despite having a strong vertebral artery, displayed intracerebral artery segmental stenosis, fewer distal branches, and poor development similar to the model group (Fig. 7e). This suggests that GXNT, particularly at a higher dosage, has a positive impact on cerebral vascular status, potentially mitigating the development of DE.

### GXNT ameliorated histopathological changes in the brain tissues in T2DM ZDF rats with DE

**Fig. 8:**
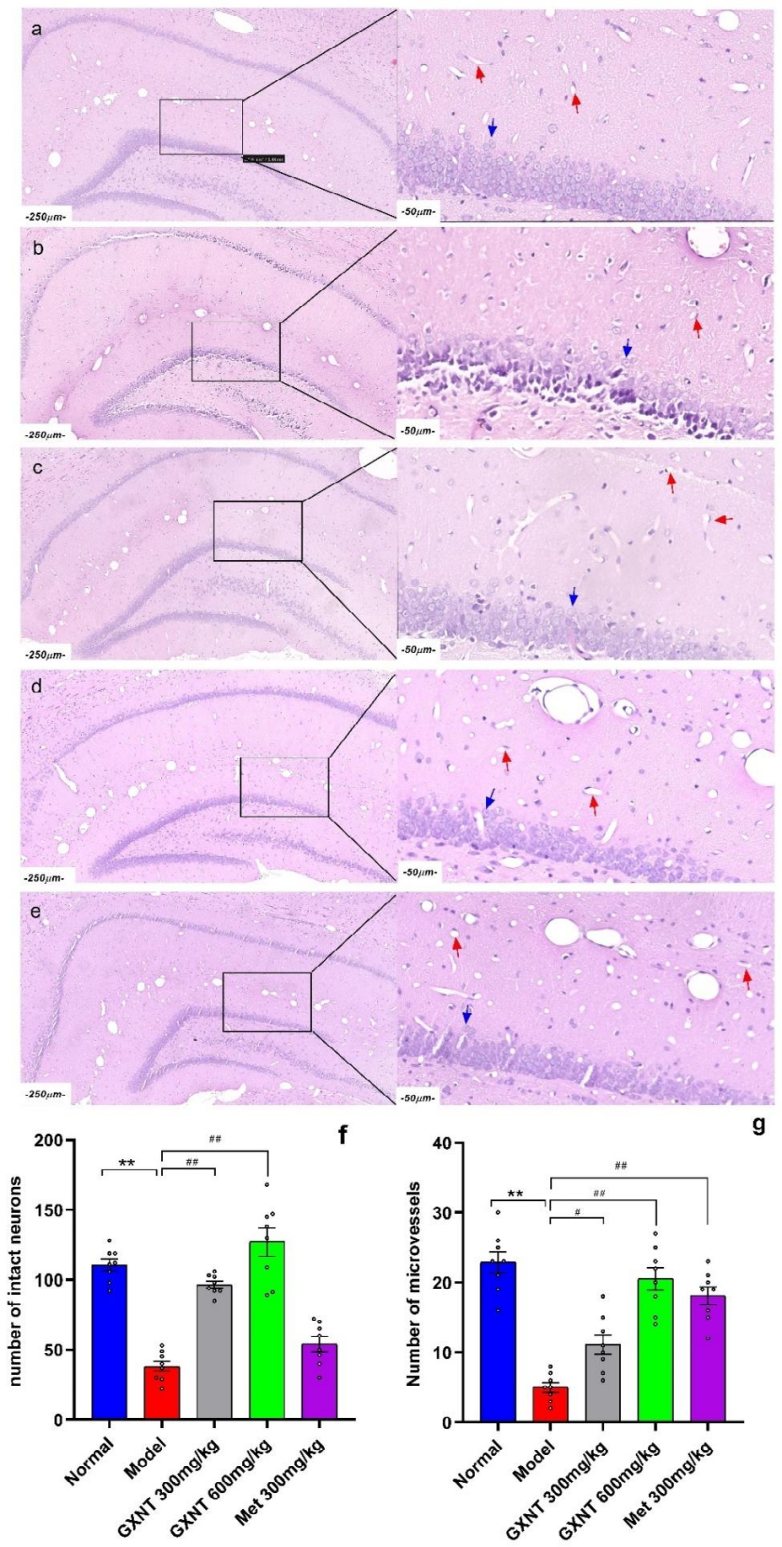
GXNT ameliorated histopathological changes in the brain tissues in T2DM ZDF rats with DE. The ZL and ZDF rats were sacrificed, and brain tissues were collected for H&E staining. In figures a-e, the scale bar on the left side of each image is 250 μm, while the right side represents the magnified region with a scale bar of 50 μm. (**a**) In the normal rat hippocampal region, numerous microvessels surround the hippocampus, with abundant neurons and normal morphology. (**b**) In the model rat hippocampal region, there are fewer neurons and microvessels surrounding the hippocampus, and a large number of neurons show pyknosis (nuclear condensation). (**c**) In the GXNT 300 mg/kg-treated rat hippocampal region, there are moderate numbers of microvessels and neurons surrounding the hippocampus, and the pyknosis of neurons is reduced, indicating improved morphology. (**d**) In the GXNT 600 mg/kg-treated rat hippocampal region, there is an increased number of microvessels and neurons surrounding the hippocampus, with a marked improvement in neuronal morphology. (**e**) The Met 300 mg/kg-treated rat hippocampal region shows a moderate number of microvessels and neurons surrounding the hippocampus. (**f**) Number of neurons in the hippocampal DG region of each experimental group of rats. (**g**) Number of microvessels in the hippocampal DG region of each experimental group of rats

Histological examination through H&E staining revealed that GXNT had a notable ameliorative effect on pathological changes in the brain tissues of DE rats. It led to an increase in the number of microvessels around the hippocampal tissue, maintenance of the normal morphology of neurons, and a reduction in neuronal pyknosis, as shown in Fig. 8a-e. The dentate gyrus (DG) region of the hippocampus was particularly examined. Quantitative analysis was conducted by counting microvessels and neurons, performed in a blinded manner. Statistical analysis using one-way ANOVA followed by Tukey’s multiple comparisons test for the number of microvessels and Welch’s ANOVA test followed by Dunnett’s T3 multiple comparisons test for the number of neurons was carried out. Results showed that compared to the normal group, the model group exhibited a significant reduction in the number of microvessels (F_4, 35_ = 29.45, p < 0.001) (Fig. 8f) and neurons (W_4.000, 16.87_ = 58.85, p < 0.001) (Fig. 8g), along with the presence of nuclear pyknosis (Fig. 8b). Treatment with 300 mg/kg and 600 mg/kg GXNT and Met increased the number of microvessels and neurons around the hippocampus and significantly reduced neuronal nuclear pyknosis (Fig. 8c, d). Compared to the model group, both the 300 mg/kg and 600 mg/kg GXNT treatment groups showed a highly significant increase in the number of microvessels and neurons (p < 0.01) (Fig. 8c, d, f, g), while the Met treatment group showed no significant change in the number of neurons (p > 0.05) but exhibited a highly significant increase in the number of microvessels (p < 0.01) (Fig. 8e, f, g). GXNT demonstrated a positive effect on histopathological changes in brain tissues, especially in the hippocampus, suggesting a potential neuroprotective role in the context of DE.

### GXNT ameliorated the ultrastructure of the cerebral cortex in T2DM ZDF rats with DE

**Fig. 9:**
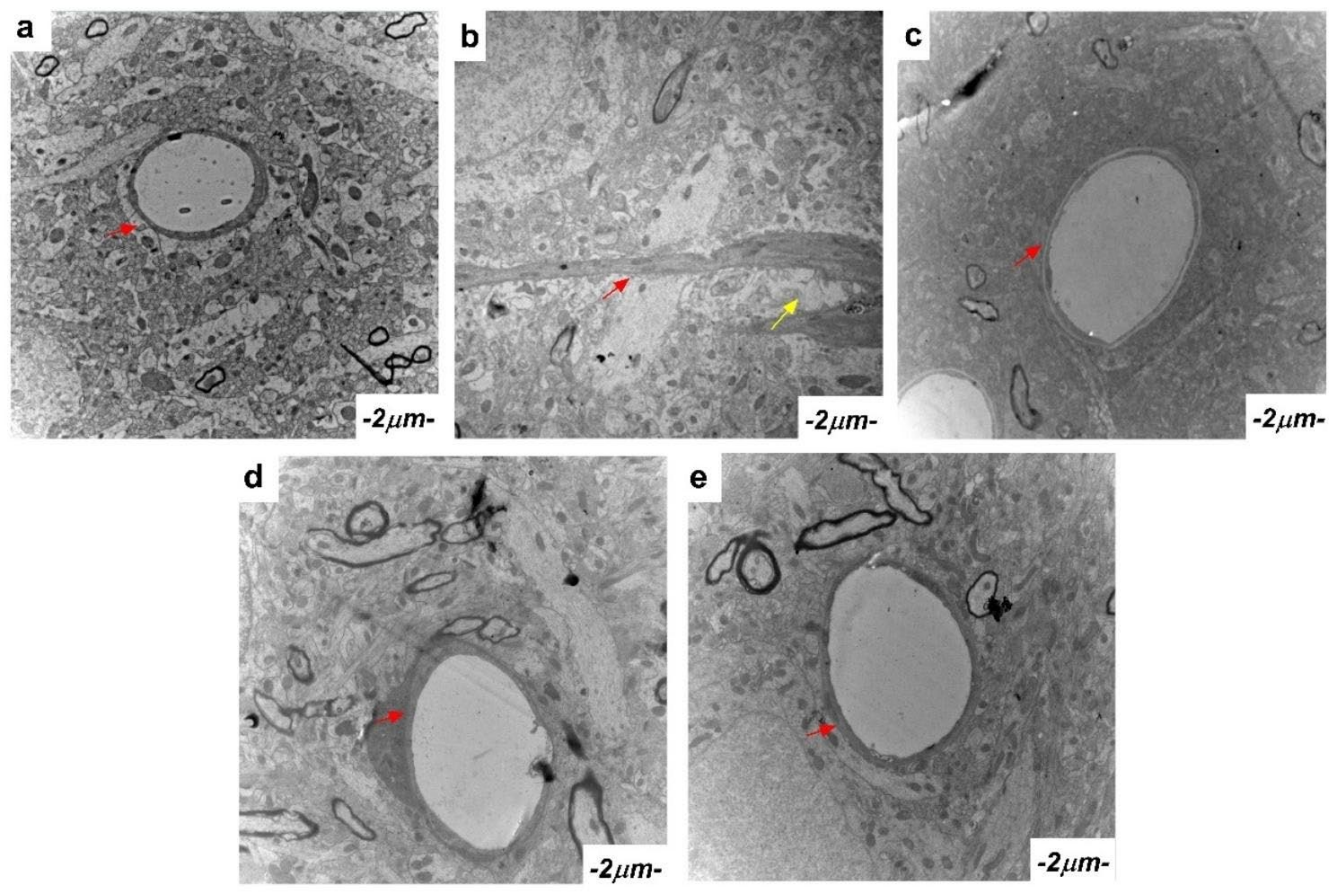
Effect of GXNT on the ultrastructure of brain tissue in T2DM ZDF rats with DE. The ultrastructure of cortical microvessels and synapses in rats was observed by transmission electron microscopy. Lead citrate solution staining (the red arrows indicate microvessels, and the yellow arrows indicate glial cells) was used to analyze the ultrastructure in each group. (**a**) shows the ultrastructure of cortical microvessels and synapses in normal rats; the microvessel lumen of the cerebral cortex is large without obvious abnormality. (**b**) shows the ultrastructure of cortical microvessels and synapses in model rats, where severe microvascular occlusion and perivascular glial cell edema occurred. (**c**) shows the ultrastructure of cortical microvessels and synapses of rats treated with GXNT 300 mg/kg, showing no occlusion of microvessels and no significant changes in perivascular cells. (**d**) shows the ultrastructure of cortical microvessels and synapses of rats treated with GXNT 600 mg/kg, showing no occlusion of microvessels and no significant changes in perivascular cells. (**e**) shows the ultrastructure of cortical microvessels and synapses of rats treated with Met 300 mg/kg, showing no occlusion of microvessels and no significant changes in perivascular cells

The ultrastructure observation of the cerebral cortex in ZDF rats revealed significant differences. The cerebral cortex microvascular lumen in the normal group appeared larger and without obvious abnormalities. Conversely, the model group exhibited severe microvascular occlusion, perivascular glial cell edema, and internal organelle degeneration. Notably, the administration of GXNT (300 mg/kg and 600 mg/kg) and Met (300 mg/kg) ameliorated these abnormalities. Microvascular occlusion was notably reduced, and there were no substantial changes in perivascular cells in the treated groups (Fig. 9). GXNT showed a positive effect on the ultrastructure of the cerebral cortex, suggesting its potential for improving cerebral vascular status and alleviating ultrastructural abnormalities associated with DE.

### GXNT ameliorated ALB and IgG leakage in the brain tissues in T2DM ZDF rats with DE

**Fig. 10:**
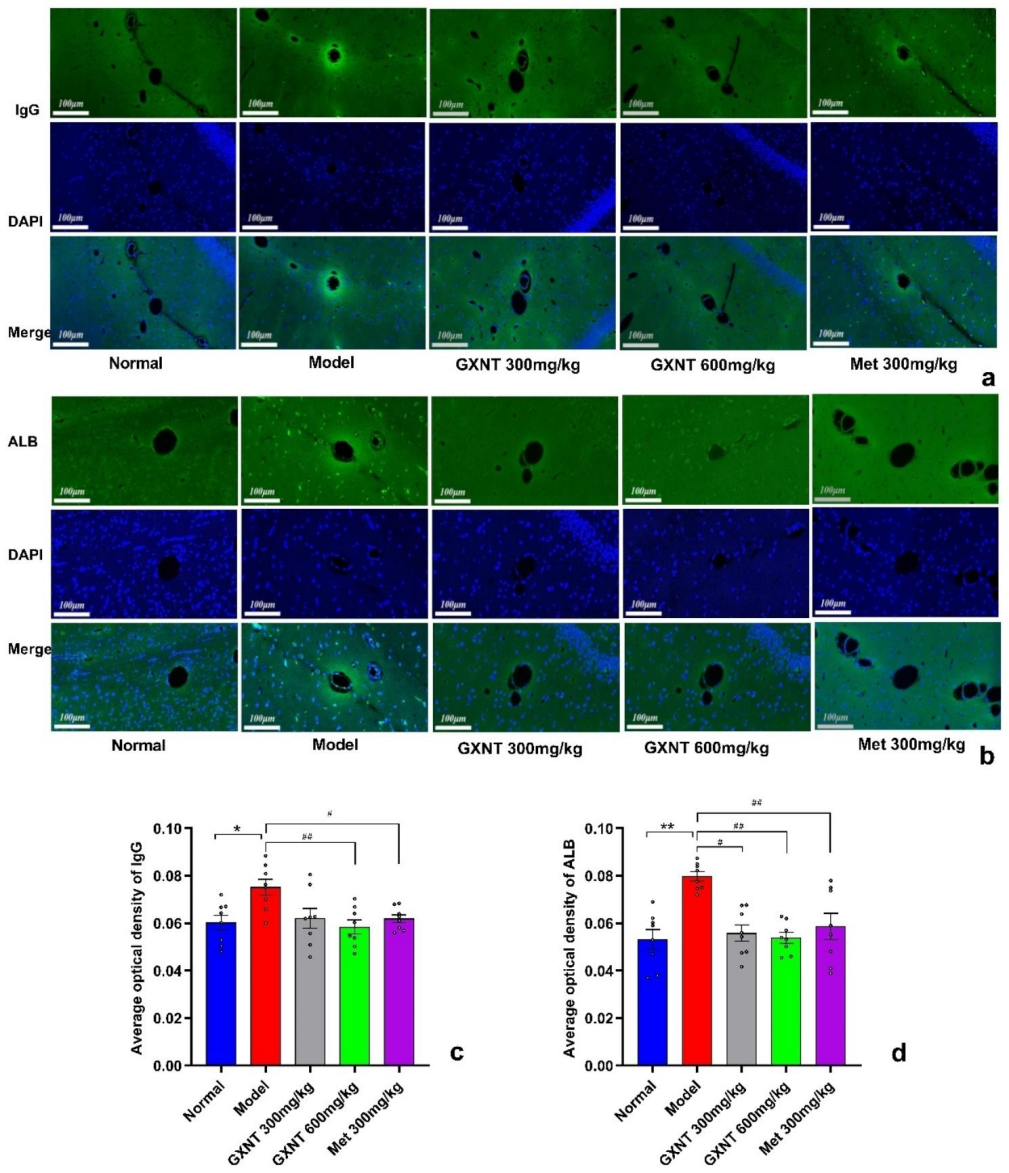
Effect of GXNT on microvascular immunofluorescence of brain tissue in T2DM ZDF rats with DE. The localization of ALB and IgG was assessed by immunofluorescence. Green fluorescence indicates ALB-stained and IgG-stained endothelial cells, and blue indicates DAPI- stained nuclei. Scale bar: 100 μm. (**a**) Representative immunofluorescence images show the expression of IgG in the indicated rats. (**b**) Representative immunofluorescence images show the expression of ALB in the indicated rats. (**c**) Quantitative analysis of the IgG area in each group. (**d**) Quantitative analysis of the ALB area in each group. GXNT, Guanxinning tablet; Met, metformin. Data are expressed as mean ± SEM, n = 8. * p < 0.05, ** P < 0.01 vs. normal group (Zucker lean control rats); #P < 0.05, ##P < 0.01 vs. model group

The immunofluorescence method was utilized to investigate ALB and IgG leakage in the cerebral cortex of ZDF rats. A one-way ANOVA followed by Tukey’s multiple comparisons test was used to analyze the fluorescence intensity of ALB, and Welch’s ANOVA followed by Dunnett’s T3 multiple comparisons test was used to statistically analyze the fluorescence intensity of IgG. Results from the analysis indicated that the model group exhibited significantly elevated fluorescence intensity of IgG (W _4.000, 16.93_ = 3.744, P < 0.05) and ALB (F _4, 35_ = 8.983, P < 0.01) in the brain vasculature surrounding area when compared to the normal group (Fig. 10a, b, c, d), indicating severe leakage of IgG and ALB from the cerebral vessels into the brain tissue. However, treatment with GXNT (300 mg/kg and 600 mg/kg) and Met (300 mg/kg) led to a notable reduction in the fluorescence intensity of IgG and ALB, implying an improvement in the leakage. Specifically, the 600 mg/kg GXNT (P < 0.01) and Met-treated (P < 0.05) groups showed a significant reduction in IgG fluorescence intensity, while the 300 mg/kg GXNT (P < 0.05), 600 mg/kg GXNT (P < 0.01), and Met-treated (P < 0.01) groups demonstrated significantly decreased ALB fluorescence intensity (Fig. 10). GXNT exhibited a mitigating effect on the leakage of ALB and IgG in the brain tissues of DE rats, indicating its potential in preserving the integrity of the blood-brain barrier (BBB) and preventing neurovascular damage.

## Discussion

The main findings of this study are as follows: (1) GXNT can improve the cognitive behavior of DE rats. (2) GXNT can regulate the glucose and lipid metabolism of DE rats, suppressing fasting blood glucose, postprandial blood glucose, and HbA1c levels, and reducing TC levels. (3) GXNT has beneficial effects on the production of endothelial factors in DE rats, increasing the levels of NO and PGI_2_ and decreasing the TXB_2_/PGI_2_ ratio. (4) GXNT improves the vascular status in the brain of DE rats, alleviating cerebral arterial obstruction and narrowing. (5) GXNT reduces the loss of neurons and microvessels in the cerebral cortex and hippocampal region of DE rats. (6) GXNT improves the blood-brain barrier function in DE rats, reducing the fluorescence density of ALB and IgG that leak from brain blood vessels into brain tissue.

According to epidemiological investigations, the prevalence of cognitive impairment in diabetic patients, known as DE, is approximately 40%, making it a significant contributor to diabetic patient mortality [39, 40]. The primary symptoms of DE include cognitive dysfunction, leading to declining learning and memory abilities, impaired language, and logical thinking; severe cases may result in an inability to perform daily activities [41, 42]. Currently, the main etiology of DE is attributed to the disturbances in glucose and lipid metabolism caused by elevated blood glucose levels, which can lead to cerebrovascular changes and an increase in BBB. permeability, allowing the entry of many large molecules into the brain and leading to neuronal damage and apoptosis. Additionally, excessive hyperglycemia can lead to the formation of advanced glycation end products by binding with proteins in the blood, which is an important factor in accelerating aging and contributing to the development of chronic degenerative diseases [43, 44]. Moreover, hyperinsulinemia resulting from insulin resistance can also contribute to cognitive impairment in DE through pathways involving β-amyloid deposition, tau phosphorylation, oxidative stress, inflammation, and synaptic plasticity, thereby increasing the risk of DE.

The ZDF rat is a spontaneously occurring type 2 diabetes rat model characterized by obesity, insulin resistance, lipid disorders, and abnormal glucose tolerance, resembling the metabolic syndrome observed in humans [45]. Microvascular changes and age-related pathologies occur in ZDF rats at an earlier stage. Studies by Oltman, C. L. investigating the vascular reactivity of the sciatic nerve epineurial arterioles have shown that vascular dysfunction is present in ZDF rats as early as 8 weeks of age, with vascular and neural complications manifesting around 12 weeks of age [46, 47]. Our previous research also identified early microvascular complications in T2DM in ZDF rats at 16 weeks of age. Notably, early-stage complications were prominent in the kidneys, retina, myocardium, and showed mild alterations in brain neurons [48]. In this study, ZDF rats were fed the Purina #5008 diet from 12 to 20 weeks of age, resulting in sustained hyperglycemia and lipid abnormalities. Imaging observations revealed cerebral arterial occlusion and poorly developed branching arteries. Histopathological examinations indicated a reduced neuronal count and nuclear condensation in the hippocampus, accompanied by a decrease in the number of microvessels. Behavioral evaluations further demonstrated impaired cognitive function, indicating the development of DE. The successful establishment of a DE animal model is crucial for our exploration of potential therapeutic agents.

Long-term abnormal blood glucose levels form the foundation for the development of DE. Research has demonstrated the regulatory impact of GXNT and its primary bioactive compounds, like salvianolic acid A, on blood glucose and lipid levels [30, 49, 50]. In this study, we also found that GXNT has an inhibitory effect on fasting blood glucose, postprandial blood glucose, HbA1c, and TC levels in DE rats. This effect is likely attributed to the abundant phenolic acids present in GXNT [51]. It’s worth noting that GXNT is a compound formulation, and the manifestation of its pharmacological activity is the result of the synergistic action of multiple components [21, 26]. This aspect is underscored in Tian et al.‘s study on mitigating cognitive dysfunction in type 2 diabetes-afflicted rats using the Huang-Lian-Jie-Du decoction [52]. The regulation of glucose and lipid metabolism may have an impact on neuronal activity. In a study focusing on Alzheimer’s disease (AD), GXNT was found to enhance neuronal metabolic activity in AD model rabbits by modulating gut microbiota and 12 different serum metabolites, including low-density lipoprotein [31]. AD represents a typical neurodegenerative ailment, and the cognitive impairment observed in DE bears resemblance to that in AD. Notably, characteristic pathological markers of AD, such as β-amyloid deposition and hyperphosphorylated tau protein leading to neurofibrillary tangles, are also evident in DE [53]. Therefore, the effective control of blood glucose and lipid levels by GXNT provides a certain basis for its neuroprotective effect on DE rats’ neurons.

Behavioral experiments are an effective means of assessing cognitive abilities and directly reflect the effectiveness of drugs in preventing and controlling DE. Rodents like to explore new environments [54]. Y-maze is a behavioral test based on the rodents’ natural curiosity for exploration. During the experiment, the animals prefer to enter the new arm of the maze (or stay for a longer period of time) rather than travel between the two training arms [55, 56]. Our experimental results showed that after treatment with different doses of GXNT, rats spent more time in the novel arm, indicating enhanced memory and exploratory abilities, suggesting that GXNT may improve cognitive impairment in rats. This further validates the beneficial effects of GXNT on DE.

The association between vascular endothelial factors and neurodegenerative diseases has been confirmed in many studies. NO is an important vasodilator and plays a neuroprotective role in neurodegenerative diseases. Research has shown that NO can alleviate inflammation, oxidative stress, and cell apoptosis, thereby protecting nerve cells from damage [57]. The balance of PGI_2_ and TXB_2_ is considered crucial in the pathogenesis and development of cerebrovascular diseases such as stroke and cerebral arteriosclerosis [58]. Studies have found that a decrease in PGI_2_ production and an increase in TXB_2_ may lead to vascular dysfunction, platelet aggregation, and thrombosis, exacerbating the condition of cerebrovascular diseases. Some studies have indicated that the imbalance of PGI_2_ and TXB_2_ is associated with cognitive impairments, such as mild cognitive impairment and preclinical Alzheimer’s disease [59]. This may be due to vascular dysfunction and changes in cerebral blood flow that lead to cognitive decline. In a study evaluating the pharmacological effects of GXNT on thrombosis in a FeCl_3_-induced rat model, it was found that GXNT significantly increased the content of 6-keto-PGF1a and regulated the TXB_2_/_6_-keto-PGF1a ratio, while it had no significant effect on TXB_2_ [22]. This further confirms that GXNT can exert an anti-thrombotic effect by modulating the activity of vascular endothelial factors. The results of this study showed that GXNT can increase NO and PGI_2_ levels and reduce the TXB_2_/PGI_2_ ratio in the DE animal model. This may be one of the reasons why GXNT plays a beneficial role in the prevention and control of cerebral microvascular narrowing or blockage in DE.

MRA is a noninvasive, magnetic resonance image-based vascular imaging technique. Angiography produces images of arteries (and, less frequently, veins) to assess their stenosis (abnormal narrowing), occlusion, aneurysm, or other abnormality [60]. The results of this study showed that GXNT can improve cerebral artery narrowing and blockage in DE rats. This was further supported by the analysis and evaluation of hippocampal microvessels and neurons in rats using H&E staining, as well as the observation of cerebral microvessels through ultrastructural examination, which visually demonstrated the beneficial effects of GXNT on DE rats. It can be said that the changes in biochemical indicators such as blood glucose, blood lipids, and vascular endothelial factors observed in the study were corroborated by the tissue and imaging findings.

ALB, an albumin produced by the liver, is the most abundant blood protein in mammals and is essential for maintaining the osmotic pressure needed for the proper distribution of fluid between blood vessels and tissues. When ALB levels change, the high pressure in the blood vessels forces more fluid into the tissue, causing lesions [61]. IgG is produced by plasma cells and is the most abundant type of immunoglobulin in serum and extracellular fluid. Under normal circumstances, IgG can cross the placental barrier but not the BBB [62]. According to ALB and IgG immunofluorescence observations, serious albumin leakage occurred in the cerebral vessels of rats in the model group, suggesting that the BBB permeability of rats increased under hyperglycemia, hyperlipidemia, insulin resistance, and other lesions, resulting in albumin passing through the BBB from the blood into the brain. Increased blood-brain barrier permeability and decreased cognitive function were associated with the severity of diabetes in diabetic animal models [63]. Through brain tissue immunofluorescence experiments, In this study, we found that GXNT reduced the fluorescence density of ALB and IgG in brain tissue, indicating the protection of the BBB and reducing the leakage of ALB and IgG from blood vessels into brain tissue. It can be seen that GXNT can, to some extent, protect the blood-brain barrier in ZDF rats, thereby preventing DE. This may be related to its effective protection of endothelial cells by influencing the levels of vascular endothelial factors.

The limitations of this study lie in the fact that while we observed promising effects of GXNT on DE in animal models and found that its regulation of glucose and lipid metabolism, as well as its potential for vascular endothelial protection, may be the underlying mechanisms by which GXNT exerts its effects on DE. However, we are aware that there are numerous factors influencing glucose and lipid metabolism, such as oxidative stress, and vascular endothelial protection involves the regulation of cytokines, enzymes, and structural proteins, such as tight junction proteins. For neurodegenerative diseases, the integrity of the BBB is particularly crucial. Therefore, we strongly recommend that future research explore the oxidative stress effects of GXNT in DE animal models and in vitro cell models, focusing on its impact on the BBB, especially regarding the tight junction proteins of vascular endothelial cells. This will further elucidate the mechanisms through which GXNT exerts its effects on DE.

## Conclusion

GXNT has a protective effect on T2DM DE rats: it can improve the cognitive behavior of DE rats, enhance the state of cerebral blood vessels, improve the morphology and quantity of neurons and microvessels in the brain tissue of rats, maintain the integrity of the BBB, and reduce the leakage of ALB and IgG from brain blood vessels into brain tissue. The regulatory effect of GXNT on glucose and lipid metabolism and its protective effect on endothelial cells may be the mechanisms by which it exerts a protective effect against DE.

### Electronic supplementary material

Below is the link to the electronic supplementary material.

Supplementary Material 1: Supplementary Fig. S1: Identification results of the main compounds in GXNT.

## Data Availability

All data generated or analyzed during this study is included in this article.

## Abbreviations

GXNT: GuanXinNing Tablet
ZDF Rats: Zucker Diabetic Fatty Rats
DE: Diabetic Encephalopathy
T2DM: Type 2 Diabetes Mellitus
Met: Metformin
TC: Total Cholesterol
TG: Triglycerides
HbA1c: Glycated Hemoglobin
NO: Nitric Oxide
TXB_2_: Thromboxane B_2_
MRA: Magnetic Resonance Angiography
DG: Dentate Gyrus
ALB: Albumin
BBB: Blood-Brain Barrier
AD: Alzheimer’s Disease
TCM: Traditional Chinese medicine
