# Supplementary material for: Protective effects of GuanXinNing tablet (GXNT) on diabetic encephalopathy in zucker diabetic obesity (ZDF) rats
Source: BMC Complement Med Ther. 2023 Oct 27;23:385. doi: 10.1186/s12906-023-04195-2 (PMC10605859; doi:10.1186/s12906-023-04195-2)
Supplement: Supplementary file 1 — Supplementary Material 1: Supplementary Fig. S1: Identification results of the main compounds in GXNT. [file 12906_2023_4195_MOESM1_ESM.docx]

**Supplementary Information：**

**Identification results of main compounds in GXNT**

The fingerprint images of Guanxingning tablets were determined using the following method.

1. Preparation of the test solution

Take 10 tablets of Guanxingning tablets for this experiment, grind them finely, mix well, weigh about 0.1g, put in a 50ml measuring flask, add 50% methanol, ultrasonic treatment (power 80W, frequency 25kHz) for 20 minutes, let cool, add 50% methanol to the scale, shake well, centrifuge (8000 rpm) for 10 minutes, take the supernatant and set aside.

2. Chromatographic conditions

HPLC: Agilent 1260, Agilent Technologies, USA

Chromatographic column: ZORBAX SB-C18 (4.6mm×250mm, 5μm)

Column temperature: 25°C Flow rate: 1.0 ml/min

Wavelength: 285nm

Mobile phase: 0.1% phosphoric acid-acetonitrile

The mobile phase gradients are shown in the following table:

| Time (minutes) | 0.1% phosphoric acid (%) | Acetonitrile (%) |
| --- | --- | --- |
| 0~10 | 97 | 3 |
| 10~30 | 97→87 | 3→13 |
| 30~35 | 87→86 | 13→14 |
| 35~60 | 86→80 | 14→20 |
| 60~90 | 80→77 | 20→23 |
| 90~100 | 77→75 | 23→25 |

3. Determination method

Aspirate 20 μl of the test solution, inject into the liquid chromatograph, determine and record the chromatogram.

4. Identify the characteristic peaks of the fingerprint profile of the test article according to the retention time against the control fingerprint profile.
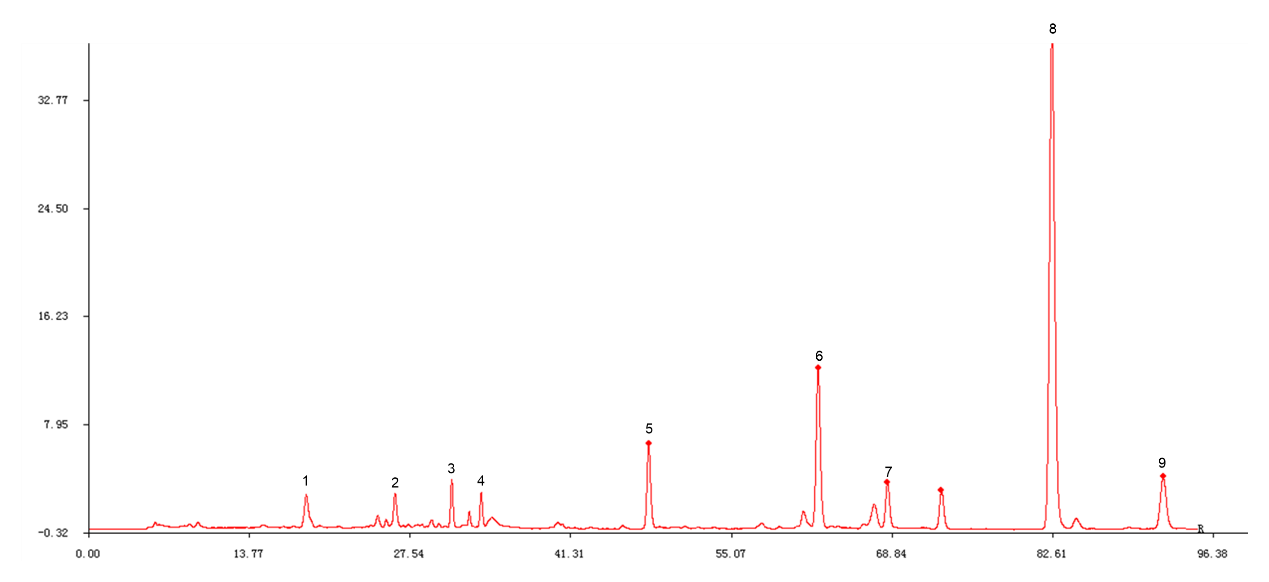


**Supplementary Fig. S1.** Fingerprint of Guanxining Tablets. (1)tanshinol, (2) protocatechualdehyde, (3) chlorogenic acid, (4) caffeic acid, (5) ferulic acid, (6) senkyunolide I (7) rosmarinic acid, (8) salvianolic acid B, and (9) salvianolic acid A.
